# Supplementary material for: Long-Term Stability Prediction for Developability Assessment of Biopharmaceutics Using Advanced Kinetic Modeling
Source: Pharmaceutics. 2022 Feb 8;14(2):375. doi: 10.3390/pharmaceutics14020375 (PMC8880208; doi:10.3390/pharmaceutics14020375)
Supplement: Supplementary file 1 [file pharmaceutics-14-00375-s001.zip › pharmaceutics-1542972-supplementary/SM_Materials-Methods-Peptide Degradation revised.pdf]

pharmaceutics

Supplementary Materials: Long-term Stability Prediction for Developability Assessment of Biopharmaceutics using Advanced Kinetic Modeling

Andreas Evers, Didier Clénet and Stefania Pfeiffer-Marek

1. Supplemental Material

1.1 Identification of chemical degradation pathways of SAR441255

1.1.1 pH dependent chemical degradation

Key degradation products of 1 mg/mL peptide in 10 mM buffers were identified using mass spectrometry and are summarized in **Table S1** as well as illustrated in **Figure S1**. The major degradation products showed different pH dependence. The major degradation product due to hydrolysis (His3-Lys39) decreased towards acidic pH values whereas oxidation and deamidation/isomerization increased at acidic pH value. Oxidation comprised mono- and di-oxidation as well as oxidation of the Trp side chain (Kynurenine).

Table S1: Key chemical degradation products after 4 weeks at 40°C in weak buffers.

Estimated amount [%]						Degradation pathway
pH 4	pH 4.5	pH 5.0	pH 5.5	pH 6.5	pH 7.4	
0.5	0.5	0.0	0.0	0.0	0.0	fatty acid side chain hydrolysis
5.2	3.9	3.5	3.1	2.3	1.3	deamidation + isomerization
2.6	3.5	4.0	5.9	5.6	4.6	hydrolysis product 3-39
2.3	2.4	2.1	2.4	0.9	0.9	other hydrolysis products
3.6	5.2	3.6	4.1	1.2	0.4	oxidation + HMWP

Figure S1: LC/UV chromatograms after 4 weeks at 40°C in weak buffers at pH values 4.0 (green), 4.5 (red), 5.0 (dark blue), 5.5 (lilac), 6.5 (black), and 7.4 (light blue). The pH values between 4.0-5.0 were stabilized using acetate buffer, pH 5.5 using succinate buffer, pH 6.5 using bis-tris buffer, and pH 7.4 using tricine buffer.

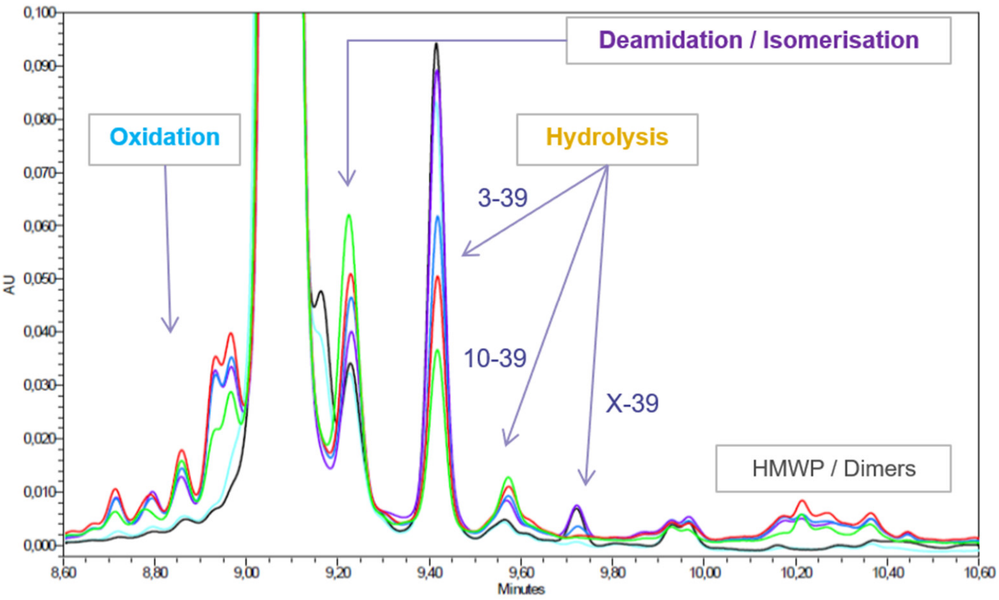

Prototype formulations containing acetate buffer, a non-ionic tonicity agent, L-methionine and metacresol showed the best chemical stability at the targeted pH value of 4.5. The addition of L-methionine greatly suppressed oxidation and the formation of HMWP (covalent dimers) at this acidic pH value. Chemical stability was strongly enhanced in prototype formulation as compared to buffer only as illustrated in **Figure S2**.

Figure S2: LC/UV chromatograms in prototype formulation at pH 4.5 that vary only in the choice of tonicity agents: glycerol (green), mannitol (red), and trehalose (dark blue). The green chromatogram corresponds to the F1 formulation in the manuscript except the lower API concentration.

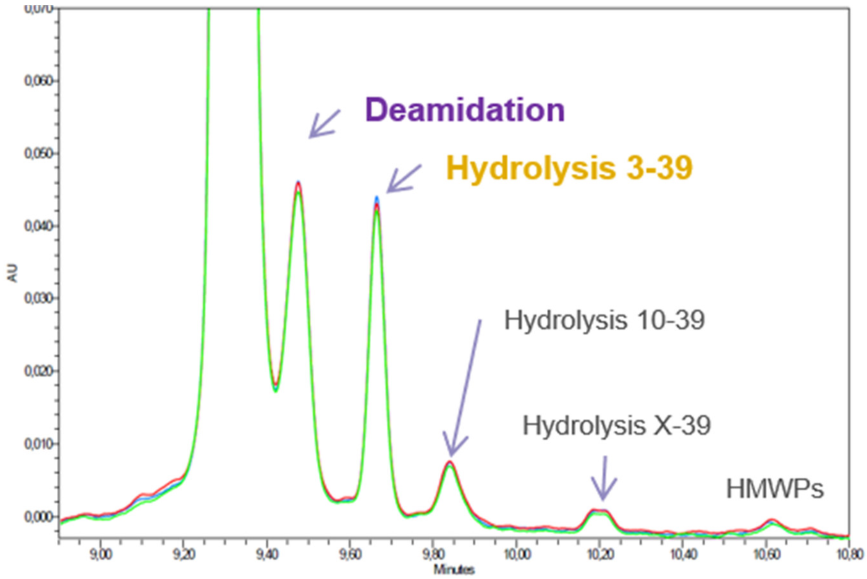

1.1.2 LC method for pH dependent stability and identification of degradation products

A Waters Acquity UPLC classic or I-class system with a photodiode-array detector (PDA) detector was used to measure the purity of the samples. The UPLC method was operated with a Waters Acquity UPLC column containing CSH C18 1.7 µm material in a 2.1 x 150 mm column dimension. The UV signal was detected at 214 nm. The flow rate was set to 0.5 mL/min with a column temperature of 50°C. Solvent A consisted of water with 500 ppm TFA and solvent B was acetonitrile with 350 ppm TFA. The gradient increases the amount of acetonitrile from 20 to 75% during 30 min (including column load and wash

phase) as summarized in **Table S2**. The injection volume was 2 µl of a 1 mg/mL sample solution at varies pH values and in various formulations.

Table S2: Gradient table for LC method to detect degradation products.

	Time [min]	Flow [mL/min]	%A	%B	Curve
1	initial	0.5	80	20	Initial
2	3	0.5	80	20	6
3	23	0.5	25	75	6
4	23.5	0.5	5	95	6
5	25.5	0.5	5	95	6
6	26	0.5	80	20	6
7	30	0.5	80	20	6
					6=linear

1.1.3 LC-MS method for identification of degradation products

An Agilent Infinity 1290 LC equipped with a diode-array detector (DAD) and hyphenated to an Agilent TOF 6230 was used to identify the degradation products. The chromatographic conditions including column were as indicated above (1.4.2) and chromatograms at 214 nm were recorded. The mass spectrometer, equipped with a dual AJS dual electrospray source, was operated according to manufacturer's instructions in the extended dynamic range modus. Spectra were recorded with positive polarity in the range of 100 to 3000 m/z at a resolution of about 15000 and using three reference masses (121, 922 and 2421) for improved mass accuracy. The mass spectrometry (MS) spectra were created and analyzed for each integrated UV-peak higher than 0.5% of the total area sum for the samples stressed for 28 days at 40°C. The results were also compared to the results of the original t(0) samples in order to discriminate between impurities (also present at t(0)) and degradation products (only present after stress).

1.2 Additional Information on Primary Packaging Material

Table S3: Supplier details for primary packaging materials used during stability studies of the peptide.

Identifier	Part	Size	Material	Supplier
PM1	container	2R iso	tubular glass, glass type I, colorless, Fiolax® CHR	Schott Schweiz AG<br>CH-9001 St. Gallen<br>Switzerland
PM1	stopper	13 mm	grey bromobutyl rubber 4023/50, Flurotec® coating (siliconization level B2-40), design 1358	West Pharmaceutical Services<br>Deutschland GmbH & Co. KG<br>Stolbergerstr. 21-41<br>52249 Eschweiler<br>GERMANY
PM1	cap	13 mm	green aluminum flanged cap BD13 with flip off; no rim; not engraved (not in contact with product)	West Pharmaceutical Services<br>Deutschland GmbH & Co. KG<br>Stolbergerstr. 21-41<br>52249 Eschweiler<br>GERMANY

PM2	cartridge	1.5 mL	tubular glass, glass type I, colorless, with flanked rim and coalesced opening at the stopper end, baked in silicon	Schott Schweiz AG<br>CH-9001 St. Gallen<br>Switzerland
PM2	cap	7.5 mm	blue alu flanged cap with inserted laminated sealing disc, isopren/bromobutyl, not siliconized	West Pharmaceutical Services<br>Deutschland GmbH & Co. KG<br>Stolbergerstr. 21-41<br>52249 Eschweiler<br>GERMANY
PM2	plunger	6 mm	grey bromobutyl rubber, siliconized 17-32 µg/mL	West Pharmaceutical Services<br>Deutschland GmbH & Co. KG<br>Stolbergerstr. 21-41<br>52249 Eschweiler<br>GERMANY

1.3 Materials Used for Manufacturing of Drug Product Solutions

Glacial acetic acid, sodium acetate trihydrate and sodium hydroxide (NaOH) were obtained from Merck (Merck Chemicals GmbH, Darmstadt, Germany), sodium chloride (NaCl) was supplied from ESCO (K+S, Hannover, Germany), glycerol 85% and metacresol were purchased from Hedinger (Aug. Hedinger GmbH & Co. KG, Stuttgart, Germany) and Polysorbate 20 (PS20) from KLK (Dr. W. Kolb Nederland B.V., Netherlands). L-methionine was obtained from Evonik (Evonik Nutrition & Care GmbH, Essen, Germany). The 15 mM acetate buffer was manufactured as 1.25-fold concentrate and used for 80% of the volume. The pH value of placebo und verum solutions was adjusted using NaOH. Polysorbate 20 was prepared as 1% dilution and used to ensure exact dosage. Manufacturing of peptide solutions was performed in a stepwise process starting with acetate buffer concentrate and followed by the API and all excipients. After dissolving all ingredients, the pH was re-adjusted using NaOH. Then, solutions were filled up with water for injection up to the final volume. Finally, solutions were filtered through 0.22 µm PVDF membrane using syringe filters (Low Protein Binding Durapore® (PVDF) Membrane, Millex VV, Merck Millipore Ltd, Tullagreen, Ireland).

1.4 Analytical Methods for Determination of Purity and HMWP

1.4.1 HPLC Method for Determination of Purity

The purpose of the analytical procedure was to monitor and quantify peptide purity by ultrahigh pressure liquid chromatography coupled to an ultraviolett detector (UPLC-UV). Stability samples were diluted to a concentration of 1 mg/mL prior to analysis. The diluent consisted of 39.96% acetonitrile, 59.94% purified water, and 0.1% trifluoroacetic acid. Peaks below a threshold of 0.1% were disregarded (limit of quantification, LOQ).

Table S4. List of reagents for peptide purity determination.

Material name and formula	Grade
Water (H2O)	Purified
Acetonitrile (CH3CN)	Gradient grade for liquid chromatography
Trifluoroacetic acid (CF3COOH)	Analytical grade

Table S5. Analytical conditions for peptide purity determination.

Stationary phase:	Acquity UPLC BEH C18 from Waters TM
Column length:	150 mm
Column internal diameter:	2.1 mm
Particle size:	1.7 µm

**Mobile phase A**	**10% acetonitrile / 90% water/ 0.1% trifluoroacetic acid**
**Mobile phase B**	**90% acetonitrile / 10% water/ 0.1% trifluoroacetic acid**
Flow rate:	0.3 mL/min
Injection volume:	5 μL
Autosampler temperature:	+5°C
Column temperature:	40°C
Detection (UV)	210 nm
Typical total run time:	35 min

Table S6. Gradient details for peptide purity determination.

Time [minutes]	Mobile phase A [%]	Mobile phase B [%]
0	70	30
3	70	30
30	40	60
30.1	0	100
32	0	100
32.1	70	30
35	70	30

1.4.2 SEC Methods for Determination of HMWP

The purpose of the analytical procedure was to quantify the amount of high molecular weight protein (HMWP) by high performance size exclusion chromatography (HP-SEC). The procedure was carried out based on the methods described in Ph. Eur. (2.2.29 Liquid Chromatography; 2.2.30 Size-exclusion chromatography) and USP (<621> chromatography) and the following instructions. Samples were diluted down to 1 mg/mL using the mobile phase. Peaks below a threshold of 0.1% were disregarded (limit of quantification, LOQ).

Table S7. List of reagents for peptide HMWP determination.

Material name and formula	Grade
Water (H2O)	Purified
Methanol (CH3OH)	Gradient grade for liquid chromatography
Trifluoroacetic acid (CF3COOH)	Analytical grade

Table S8. Analytical conditions for peptide HMWP determination.

**Stationary phase:**	**Sepax Zenix SEC, 100 Å, 3 μm or equivalent**
Column length:	300 mm
Column internal diameter:	7.8 mm
Column material:	Stainless steel
**Mobile phase:**	**59.94% Methanol / 39.96% water/ 0.1% trifluoroacetic acid**
Procedure:	0-25min / 100% mobile phase
Flow rate:	0.7 mL/minute
Injection volume:	8 μL
Autosampler temperature:	Set autosampler temperature at +5°C±3°C
Column temperature:	Set column thermostat temperature at +65°C
Detection:	215 nm (UV)

Table S9. Approximate retention times for peptide HMWP determination.

Substance	Approx. retention times
Peptide HMWP-Dimer 1	8.4 min
Peptide HMWP-Dimer 2	8.9 min
Peptide	10.0 min

Calculations

The sum of all peak areas of the signals including the peptide and the areas of the HMWPs (dimer 1 and dimer 2) is determined. The content of proteins with high molecular weight (HMWPs) is calculated as follows:

$$\frac{\sum A_{HMWP}}{\sum A_{Total}} \cdot 100 = Amount[\%]$$

Amount = Content of proteins with high molecular weight [Area %]
Σ A_{HMWP} = Sum of all peak areas of HMWPs
Σ A_{Total} = Sum of the peak areas of HMWPs and peptide
